# Limited Evidence for a Relationship between HIV-1 Glycan Shield Features in Early Infection and the Development of Neutralization Breadth

**DOI:** 10.1128/JVI.00797-21

**Published:** 2021-08-10

**Authors:** Yifan Li, Hongjun Bai, Eric Sanders-Buell, Vincent Dussupt, Samantha Townsley, Gina Donofrio, Meera Bose, Anne Marie O’Sullivan, Hannah Kibuuka, Lucas Maganga, Sorachai Nitayaphan, Josphat Kosgei, Punnee Pitisuttithum, Supachai Rerks-Ngarm, Leigh Anne Eller, Nelson L. Michael, Merlin L. Robb, Julie Ake, Sandhya Vasan, Sodsai Tovanabutra, Shelly J. Krebs, Morgane Rolland

**Affiliations:** a U.S. Military HIV Research Program, Walter Reed Army Institute of Research, Silver Spring, Maryland, USA; b Henry M. Jackson Foundation for the Advancement of Military Medicine, Inc., Bethesda, Maryland, USA; c Makerere University Walter Reed Project, Kampala, Uganda; d National Institute for Medical Research–Mbeya Medical Research Center, Mbeya, Tanzania; e Armed Forces Research Institute of Medical Sciences, Bangkok, Thailand; f Kenya Medical Research Institute /U.S. Army Medical Research Directorate-Africa/Kenya–Henry Jackson Foundation MRI, Kericho, Kenya; g Mahidol University, Bangkok, Thailand; h Thai Ministry of Public Health, Nonthaburi, Thailand; i Center for Infectious Disease Research, WRAIR, Silver Spring, Maryland, USA; Ulm University Medical Center

**Keywords:** Env glycans, HIV-1, evolution, neutralization

## Abstract

Identifying whether viral features present in acute HIV-1 infection predetermine the development of neutralization breadth is critical to vaccine design. Incorporating such features in vaccine antigens could initiate cross-reactive antibody responses that could sufficiently protect vaccinees from HIV-1 infection despite the uniqueness of each founder virus. To understand the relationship between Env determinants and the development of neutralization breadth, we focused on 197 individuals enrolled in two cohorts in Thailand and East Africa (RV144 and RV217) and followed since their diagnosis in acute or early HIV-1 infection. We analyzed the distribution of variable loop lengths and glycans, as well as the predicted density of the glycan shield, and compared these envelope features to the neutralization breadth data obtained 3 years after infection (*n*  = 121). Our study revealed limited evidence for glycan shield features that associate with the development of neutralization breadth. While the glycan shield tended to be denser in participants who subsequently developed breadth, no significant relationship was found between the size of glycan holes and the development of neutralization breadth. The parallel analysis of 3,000 independent Env sequences showed no evidence of directional evolution of glycan shield features since the beginning of the epidemic. Together, our results highlight that glycan shield features in acute and early HIV-1 infection may not play a role determinant enough to dictate the development of neutralization breadth and instead suggest that the glycan shield’s reactive properties that are associated with immune evasion may have a greater impact.

**IMPORTANCE** A major goal of HIV-1 vaccine research is to design vaccine candidates that elicit potent broadly neutralizing antibodies (bNAbs). Different viral features have been associated with the development of bNAbs, including the glycan shield on the surface of the HIV-1 Envelope (Env). Here, we analyzed data from two cohorts of individuals who were followed from early infection to several years after infection spanning multiple HIV-1 subtypes. We compared Env glycan features in HIV-1 sequences obtained in early infection to the potency and breadth of neutralizing antibodies measured 1 to 3 years after infection. We found limited evidence of glycan shield properties that associate with the development of neutralization breadth in these cohorts. These results may have important implications for antigen design in future vaccine strategies and emphasize that HIV-1 vaccines will need to rely on a complex set of properties to elicit neutralization breadth.

## INTRODUCTION

Many enveloped viral pathogens use the host cell glycosylation machinery to cover the surface of their proteins with self-glycans. Glycosylation was shown to serve key functions for different viral proteins, such as the hemagglutinin of influenza virus (1), the coronavirus spike (2), the glycoprotein complex of Lassa virus (3), or the glycoproteins of Ebola (4, 5), dengue (6), and Zika (7) viruses or HIV-1. The structure and function of HIV-1’s Envelope (Env) glycan shield has been intensely studied. The HIV-1 Env on mature virions form a trimer of noncovalently associated gp120-gp41 heterodimers. Glycans densely cover the Env surface with medians of 25 glycans found on gp120 and 4 glycans found on gp41 (8). Hence, about half of the mass of HIV-1 viruses is constituted by glycans covering Env (9). This poses a significant challenge for vaccine design since these self-glycans derived through the host cell pathways are poorly immunogenic. However, licensed vaccines typically try to mimic native antigens. Because eliciting responses against nonnative glycoforms or against conserved epitopes that are occluded on mature Env would appear counterproductive, HIV-1 vaccine strategies try to integrate knowledge of HIV-1 glycosylation.

While the glycan shield has long been recognized for its ability to mask the viral Env from the immune system and to confer escape from the host’s neutralizing antibodies, there are now multiple examples of broadly neutralizing antibodies (bNAbs) capable of recognizing glycans. Such bNAbs were shown to include glycans in their epitopes, recognize complex glycans, or penetrate the glycan shield through long heavy-chain complementarity-determining region-3 (HCDR3) regions that allow protein/protein contacts (e.g., PGT128/145/151) (10–14).

Approximately half of HIV-1-infected individuals develop antibodies that can neutralize at least half of the viruses in large panels of diverse viruses, and around 20% of individuals mount bNAbs (15–18). While the induction of bNAbs by a subset of HIV-1-infected individuals does not seem to be beneficial in terms of disease progression for these individuals, it is widely accepted that an HIV-1 vaccine would need to elicit such bNAbs to be protective against the myriad HIV-1 circulating viruses. Since generation of bNAbs in humans has proven challenging to date, manipulation of glycans on HIV-1 vaccine antigens might improve bNAb induction *in vivo*. Previous studies highlighted the complexity of the interactions between glycans and the development of neutralization breadth. Different reports emphasized that Env with shorter loops (specifically V1V2) and thus a limited number of glycans may be selected upon transmission to a new host and associated with increased sensitivity to neutralization (19–23). However, at the population level, there was no strong evidence for the selection of shorter loops or fewer potential N-linked glycosylation sites (PNGS), and circulating viruses appear to show an increase in resistance to antibody neutralization since the beginning of the epidemic (24–29).

More recent studies have sought to integrate additional information pertaining to the trimeric Env structure in addition to sequence characteristics such as loop length. Based on Env sequences and neutralization breadth information from 12 individuals followed for several years, Wagh et al. showed that an intact or complete glycan shield at the beginning of infection was associated with the development of neutralization breadth (30), whereas multiple reports described how holes in the glycan shield generate strain-specific neutralizing antibody responses (31–34). The results presented above illustrate a multifaceted understanding of the role of glycans in the development of neutralization breadth. Determining viral features, such as glycan features, in the early stages of HIV-1 infection in individuals who develop neutralization breadth may be crucial to understanding the steps that a vaccine regimen would seek to replicate in order to elicit similar neutralization breadth. In this study, we sought to evaluate this hypothesis by focusing on 197 individuals from two cohorts in East Africa and Thailand (RV217 and RV144) who were followed since their diagnosis in acute or early HIV-1 infection to determine the potential role of glycans on founder and early HIV-1 Envs. Participants enrolled in the RV217 prospective cohort were tested for HIV-1 infection twice a week (35), while participants in the RV144 vaccine efficacy trial were tested every 6 months (36). HIV-1 genomes were sequenced via endpoint dilution from plasma samples collected at HIV-1 diagnosis and at later time points (37, 38). We characterized HIV-1 Env sequences by evaluating the distribution of variable loop lengths and glycans, as well as the predicted density of the glycan shield, and compared these envelope features to the neutralization breadth data from these participants (39, 40). Our study revealed limited evidence of glycan shield features that associate with the development of neutralization breadth in these cohorts.

## RESULTS

### Env sequences from 197 acute and early HIV-1 infections.

We analyzed >4,000 Env sequences collected from 197 individuals who became HIV-1 infected while enrolled in the prospective RV217 study (*n* = 87) or the RV144 vaccine efficacy trial (*n* = 110, 44 vaccine and 66 placebo recipients). RV217 participants were tested for HIV-1 RNA twice weekly allowing diagnosis early in the infection (35, 38). All participants had *env* sequences sampled in the first month after HIV-1 diagnosis (range, 0 to 29 days). Forty-three participants had sequences derived at three time points in the first 6 months of infection: at about 1 week (0 to 16 days), 1 month (18 to 48 days) and 6 months (132 to 261 days) after diagnosis. RV144 participants were tested for HIV-1 infection only every 6 months; hence, their sequences were derived from samples from early infection, obtained after individuals had already seroconverted (36, 37). We analyzed 688 Env sequences obtained at HIV-1 diagnosis from 110 RV144 participants. Five to 10 Env sequences per subject (extracted from SGA-derived HIV-1 near-full-length genome sequences) were used to calculate the mean number of PNGS, loop lengths, or the net charge at each time point.

**FIG 1:**
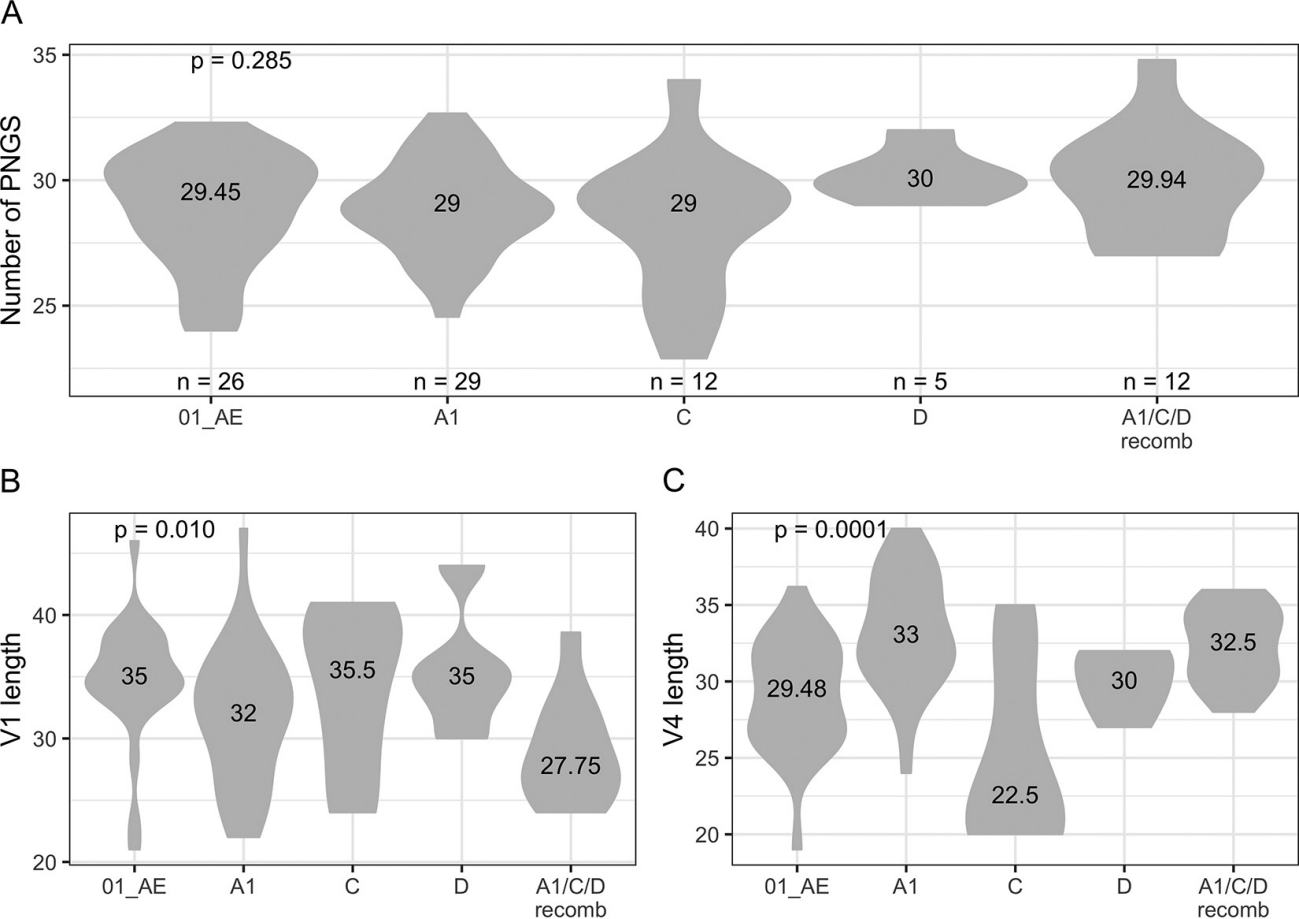
Similar numbers of PNGS found across HIV-1 subtypes. (A) Numbers of PNGS found in Env sequences from RV217 participants. (B and C) Loop lengths for V1 (B) and V4 (C), represented because significant differences were observed across subtypes. The figure shows the number of participants and median values for each group along with *P* values for Kruskal-Wallis tests.

RV217 participants were diagnosed in Kenya, Tanzania, Uganda, and Thailand and were infected by different HIV-1 subtypes: A1, C, D, and their recombinants (A1/C/D recombinants) in East Africa and CRF01_AE in Thailand (Fig. 1). We found that the number of PNGS in Env did not vary across subtypes (*P* = 0.148, Fig. 1A). While the total number of PNGS can vary by more than 10 glycans, the median corresponded to 29 (subtypes A1 and C) or 30 (subtype D, CRF01_AE and A1/C/D recombinants from East Africa) PNGS in our cohort. Across subtypes, some differences could be seen when looking at the loop lengths for V1 and V4 (Kruskal-Wallis *P* ≤ 0.003, Fig. 1B and C). For example, the median V1 length varied between 28 amino acids (aa) for A1/C/D-containing recombinants and 36 aa for subtype C, while the median V4 length varied between 23 aa for subtype C and 33 aa for subtype A1. The 110 RV144 participants included in this study were infected with CRF01_AE viruses in Thailand. Similar to RV217 participants, the median number of PNGS was 28 (interquartile range [IQR], 27 to 30), and there was no significant difference between RV144 vaccine and placebo groups (*P* = 0.805). Finally, whether an HIV-1 infection is established by single or multiple founders is an important factor in acute infection (41, 42), and yet we found no evidence that founder multiplicity was affecting glycan characteristics in RV217 participants (median number of PNGS = 29.14 [multiple] and 29 [single], *P* = 0.915) or RV144 participants (vaccine, median number of PNGS = 28.91 [multiple] and 28 [single], *P* = 0.449; placebo, median number of PNGS = 27.93 [multiple] and 29 [single], *P* = 0.112).

### No glycan variation in the first year of infection.

**FIG 2:**
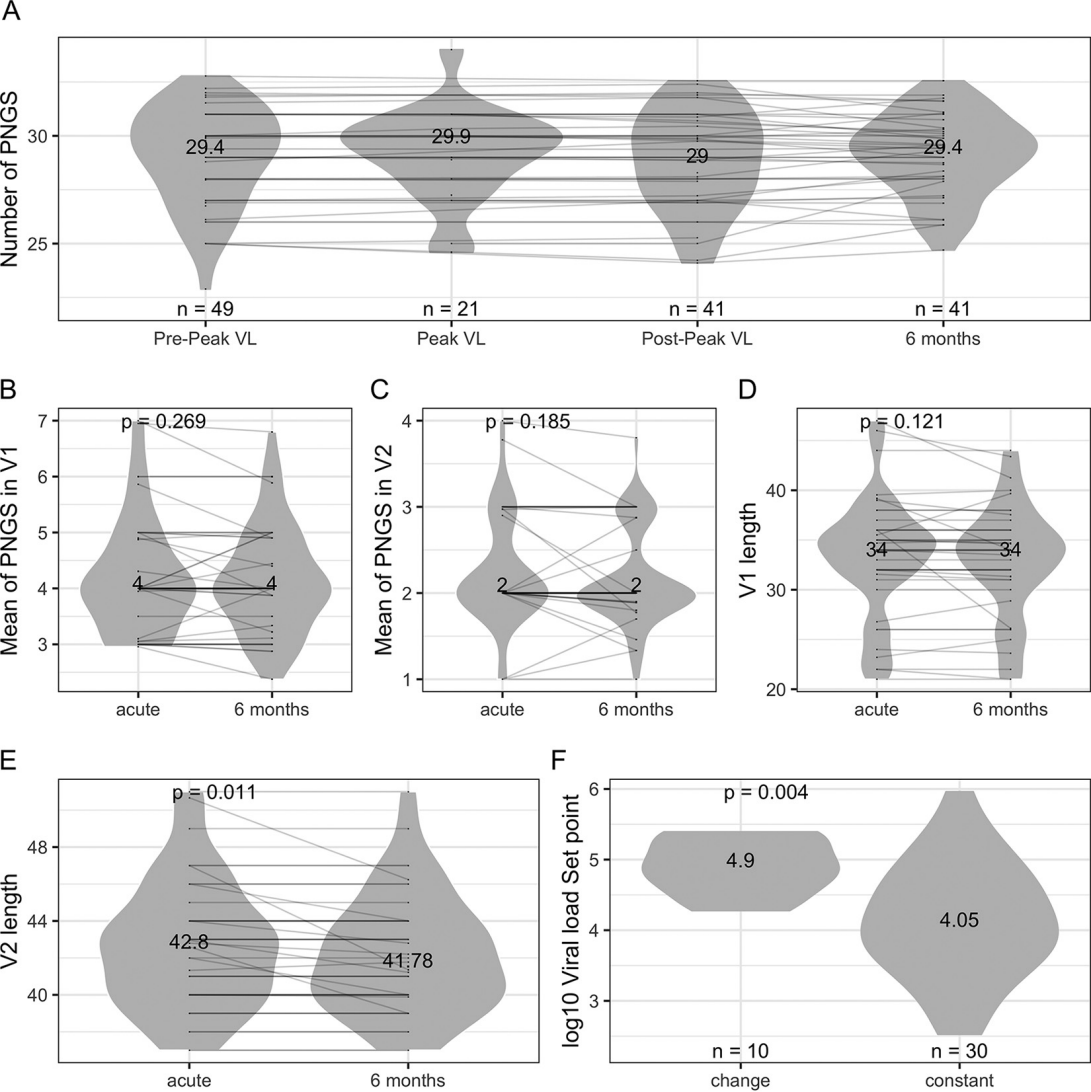
Numbers of PNGS and loop lengths over the first 6 months of infection. (A) The plot compares the number of PNGS in Env sequences sampled before peak viremia (0 to 15 days postdiagnosis), at peak (0 to 25 days postdiagnosis), about 1 month after diagnosis (postpeak, 21 to 42 days postdiagnosis), and 6 months after diagnosis (132 to 261 days). The figure shows the number of participants and median values for each group. (B to E) The numbers of PNGS in V1 (B) and V2 (C) and the V1 length (D) and V2 length (E) were compared to sequences sampled during acute infection and 6 months later. The figures show the median values for each group along with *P* values for Wilcoxon signed-rank test. (F) Set point viral load was compared between individuals who showed changes in V2 length over the first 6 months of infection and those whose V2 sequences showed no variation. The figure shows median values for each group, along with *P* values for Mann-Whitney tests.

We grouped all sequences irrespective of the corresponding subtype/CRF since these were not linked to differences. Using all sequences sampled within 42 days of HIV-1 diagnosis in RV217 participants, there was an average 29 PNGS per sequence (range, 23 to 35). As expected, most of the variation in loop lengths was found in the variable loops V1, V2, V4, and V5: the mean loop lengths were 33 aa for V1 (range, 21 to 47), 42 aa for V2 (range, 37 to 51), 31 aa for V4 (range, 19 to 40), and 15 aa for V5 (range, 12 to 24). There was no variation for V3 (mean, 35; range, 34 to 35; Table 1). Since we had a large number of sequences sampled in acute infection in RV217 participants, we partitioned these obtained before peak viremia, at peak viral load (VL), or 1 month after diagnosis. The number of PNGS per sequence remained constant (*n* = 29) at these early time points, as well as at 6 months after diagnosis, reflecting that loop lengths were as likely to extend or shorten depending on the individuals (Fig. 2A). Since the overall variation in Env is usually driven by differences in V1 and V2 loops, we analyzed these loops separately: the summary numbers remained constant over time for V1 (4 PNGS, *P* = 0.269; 34 aa, *P* = 0.121) and for V2 (2 PNGS, *P* = 0.185) (Fig. 2B to D). However, for V2 loop lengths, there was a significant difference between acute infection and 6 months, driven by a small decrease in V2 length in some individuals (median = 42.8 [acute] versus 41.78 [6 months], *P* = 0.011) (Fig. 2E). A total of 9 individuals showed a decrease in V2 length (by 0.11 to 4.4 aa), and 1 showed an increase by 0.45 aa, while 30 individuals showed no variation. Interestingly, individuals for whom we identified changes in V2 length showed higher set point viral load than those with V2 that remained constant: SPVL = 4.9 versus 4.05 (*P* = 0.004) (Fig. 2F).

**TABLE 1:** Characteristics of HIV-1 glycan and loop lengths in RV217 and RV144 participants^*a*^

Group and parameter	Env PNGS	V1 PNGS	V1 length	V2 PNGS	V2 length	V3 PNGS	V3 length	V4 PNGS	V4 length	V5 PNGS	V5 length
RV217, acute											
Min.	22.89	2	21	0	37	0	34	2	19	0	12
Q1	27.99	3.25	28.67	2	40	1	35	4	28	1	13.6
Median	29	4	33.87	2	42	1	35	4	31	1	14
Mean	29.07	4.23	32.96	2.2	42.41	0.98	34.83	4.28	30.48	1.43	14.76
Q3	30.58	5	37	2.5	44	1	35	5	33	2	15
Max.	34.8	8	47	4	51	1	35	6.94	40	3	24
											
RV217, 6 mo											
Min.	24.7	2.38	21	1	37	0.95	34	2	20	0.1	10.4
Q1	28	3.5	31	1.9	40	1	35	3.89	28.05	1	14
Median	29.4	4	34	2	41.78	1	35	4.88	31	1.88	14
Mean	29.12	4.15	32.89	2.17	42.18	1	34.85	4.39	30.81	1.52	14.71
Q3	30.3	4.9	36	2.5	44	1	35	5	33	2	15
Max.	32.56	6.8	44	3.8	51	1	35	6.33	38	2.7	22.07
											
RV144											
Min.	23	2	20.5	0	37	0	33.83	1.17	20	0.8	12
Q1	27.07	4	33	1.88	40	1	35	3	26	1	13.94
Median	28.25	5	35	2	40.5	1	35	4	28	2	15
Mean	28.47	4.68	35.15	2.05	42.12	0.88	35.06	3.68	27.67	1.7	15.2
Q3	29.93	5	37.19	2	44.2	1	35	4	29.81	2	16
Max.	34.8	6.67	47	4.9	61	1	36	6	36.78	3	28

a Summary measures of the number of PNGS and loop lengths (expressed as the numbers of amino acids) are provided for HIV-1 sequences from RV217 and RV144 participants. Sequences from RV217 participants were sampled in the first month after HIV-1 diagnosis and 6 months later, sequences from RV144 were obtained within the first 6 months of infection. Min., minimum; Max., maximum.

Next, we investigated whether loop length or glycan characteristics had an impact on markers of disease progression. We found no relationship between set point viral load and the number of PNGS in acute infection or at 6 months (acute, Rho [ρ] = −0.05 and *P* = 0.688; 6 months, Rho = −0.03 and *P* = 0.834). Likewise, there was also no association with viral loads measured at peak or at the nadir following peak nor when considering loop lengths instead of the number of PNGS (*P* ≥ 0.593). We also ran these comparisons separately for each variable loop; there was no robust evidence of an association, except for a few cases. For example, the length of V1 at peak viremia was negatively associated with peak viral loads (Rho = −0.59, *P* = 0.005), but the number of PNGS in V1 prepeak was associated with peak VL (Rho = 0.33, *P* = 0.022), whereas the length of V4 at 6 months postinfection was negatively associated with set point loads (Rho = −0.48, *P* = 0.002).

We showed in the RV217 data set that there was no overall change in the number of PNGS in the first 6 months of infection (Fig. 2); thus, we analyzed all RV144 sequences together since these were collected within 6 months of infection. When we analyzed the data from 110 RV144 participants, we also found no relationship between glycan features and set point viral load (Spearman correlation coefficient Rho = 0.082, *P* = 0.514). Results were similar when analyzing vaccine and placebo groups separately (vaccine, Rho = 0.17 and *P* = 0.444; placebo, Rho = 0.005 and *P* = 0.976).

### No longitudinal variation over calendar years.

**FIG 3:**
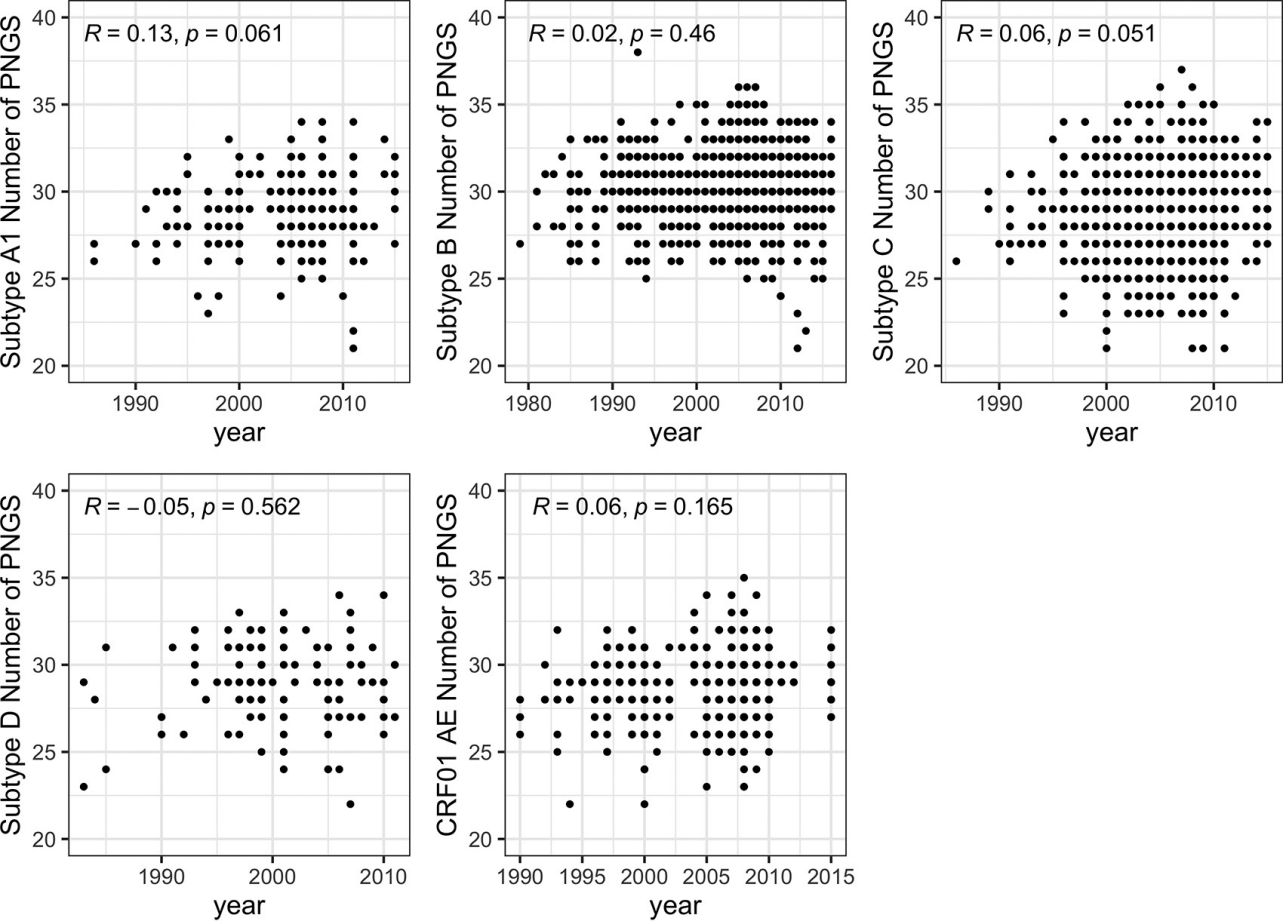
No variation in the number of PNGS in Env sequences sampled since the beginning of the epidemic. The relationship between numbers of PNGS and time was calculated using Spearman’s correlations. Similar patterns were seen across HIV-1 subtypes. The number of independent sequences sampled per subtype varied between 116 (subtype D) and 1184 (subtype C) sequences. The figure shows Spearman’s ρ and *P* values.

Sequences from the RV217 and RV144 cohorts were sampled over a limited time frame (RV217, 2009 to 2014; RV144, 2004 to 2009) relative to the duration of the HIV-1 epidemic. We evaluated how sequences from this time period related to publicly available sequences sampled since the beginning of the epidemic. Large sets of Env sequences were analyzed to retain only those that were independent, not hypermutated and corresponding to a complete open reading frame. This resulted in data sets of Env sequences from subtypes A1 (203 sequences), B (1,035 sequences), C (1,184 sequences), D (116 sequences), and CRF01_AE (497 sequences). As would be expected, the extremes of the distribution tended to increase over time. Yet, we found no correlation between the number of PNGS and the year of sampling: Rho values ranged between −0.05 and 0.13, with no significant *P* values, and there was no indication of a trend toward an increase or a decrease in the number of PNGS over time (Fig. 3).

**FIG 4:**
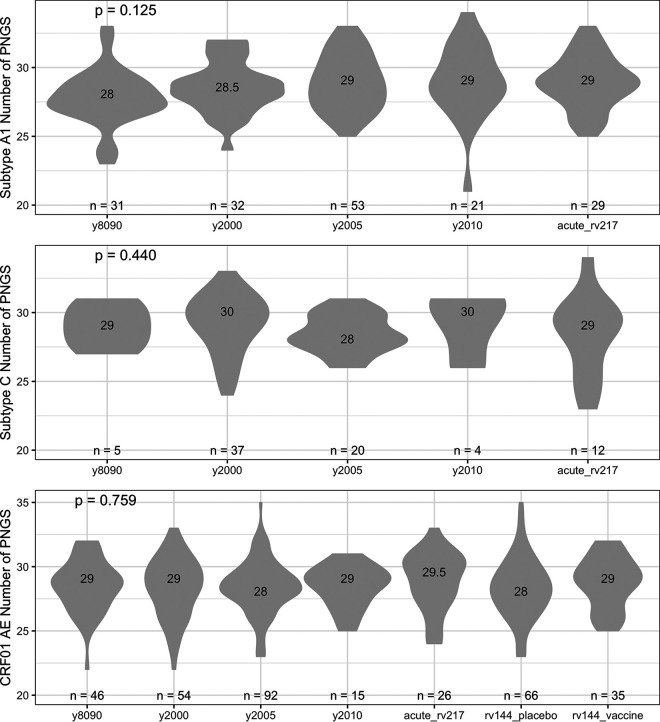
No variation in the number of PNGS found in Env since the beginning of the epidemic. The median number of PNGS found in circulating Env sequences was compared over time and across subtypes. Data sets of circulating sequences were compared to the RV217 and RV144 cohorts; sequences from RV144 vaccine and placebo recipients were analyzed separately. The figure shows the number of participants and median values for each group, along with *P* values for Kruskal-Wallis tests.

Since our RV217 and RV144 sequences were sampled from four countries, we restricted our data sets to sequences from those countries and considered sequences from Kenya, Tanzania, and Uganda together and those from Thailand separately. Sequences were grouped by time periods (those sampled in the 1980s and 1990s and those sampled around 2000, 2005, and 2010). For subtype A1, the number of sequences in each group of circulating sequences ranged between 21 and 53 and the median number of PNGS varied between 28 (in the 1980s and 1990s) and 29 (around year 2005 and 2010) over time, which is equivalent to the 29 PNGS identified in RV217’s A1 sequences (Kruskal-Wallis, *P* = 0.125) (Fig. 4). Patterns were similar for subtype C or CRF01_AE (Kruskal-Wallis, *P* = 0.440 and 0.759, respectively) (Fig. 4); there were too few subtype D sequences for this comparison.

**FIG 5:**
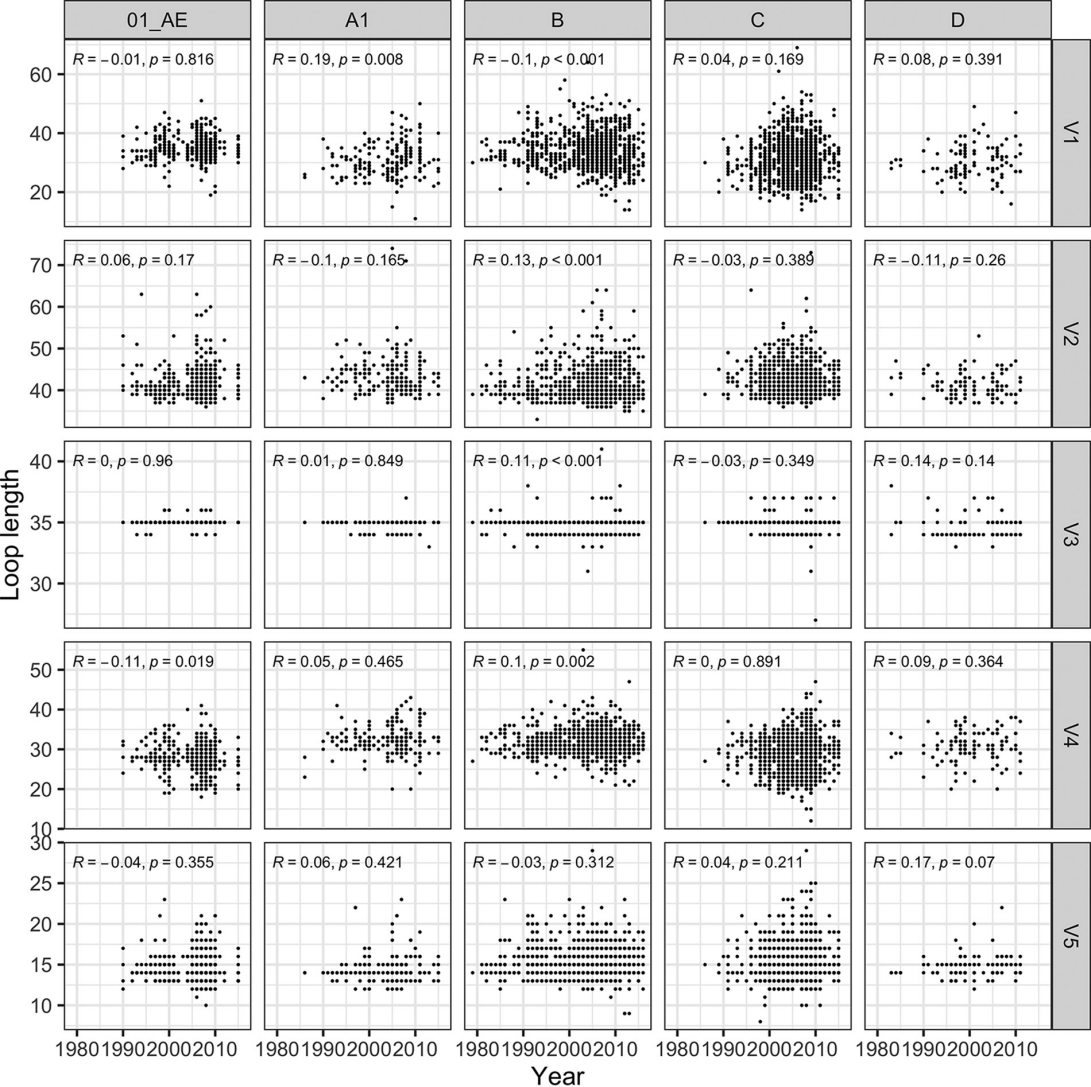
Little variation in the number of PNGS found in Env since the beginning of the epidemic. The relationship between loop lengths and time was calculated using Spearman’s correlations across HIV-1 subtypes and CRF. The figures show Spearman’s ρ and *P* values.

Parallel analyses of loop lengths showed that all loops (except V3 which usually consists of 35 aa) became more variable across individuals over time; however, there was no significant trend toward an increase or a decrease in the loop lengths over time (Fig. 5). Significant differences observed in Table 2 were associated with large variations in the number of sequences available for comparison at each time period.

**TABLE 2:** Characteristics of HIV-1 glycan and loop lengths in circulating sequences^*a*^

Subtype	Env PNGS	V1 PNGS	V1 length	V2 PNGS	V2 length	V3 PNGS	V3 length	V4 PNGS	V4 length	V5 PNGS	V5 length
Subtype A1											
y8090	28	4	29	2	43	1	35	4	32	1	14
y2000	29	4	29	2	42	1	35	5	32	2	14
y2005	29	4	32	2	42	1	35	5	32	1	14
y2010	29	4	31	2	42	1	35	5	33	1	14
K-W *P*	0.317	0.038	0.031	0.886	0.360	0.161	0.583	0.999	0.763	0.370	0.679
											
Subtype B											
y8090	30	4	34	2	39	1	35	4	31	1	15
y2000	31	4	35	2	40	1	35	4	31	2	15
y2005	31	4	34	2	40	1	35	5	32	2	15
y2010	30	4	33	2	41	1	35	4	32	2	15
K-W *P*	0.315	0.333	0.009	0.0005	<0.0001	0.088	<0.0001	0.037	0.001	0.012	0.283
											
Subtype C											
y8090	29	4	29.5	2	42	1	35	4	29	1	15
y2000	29	4	31.5	2	41	1	35	4	28	1	15
y2005	29	4	31	2	42	1	35	4	28	1	15
y2010	29	4	31	2	42	1	35	4	29	1	15
K-W *P*	0.047	0.063	0.003	0.602	0.209	0.644	0.004	0.186	0.001	0.007	0.715
											
Subtype D											
y8090	29	4	28	2	41	1	34	4	31	2	14
y2000	29	4	34	2	40	1	34	4	31	2	15
y2005	28	3	29	2	40	1	34.5	4	30.5	2	15
y2010	28	4	29.5	2	39.5	1	34.5	5	32.5	1	14
K-W *P*	0.457	0.017	0.022	0.220	0.452	0.788	0.226	0.356	0.701	0.576	0.334
											
Subtype CRF01_AE											
y8090	29	5	34	2	40	1	35	4	28	2	14
y2000	29	5	34	2	41	1	35	4	28	2	14
y2005	29	5	35	2	41	1	35	4	28	2	14
y2010	29	5	34	2	41	1	35	4	27	2	14
K-W *P*	0.813	0.363	0.263	0.641	0.320	0.018	0.210	0.282	0.022	0.315	0.920

a K-W *P*, Kruskal-Wallis *P* value. Median values for loop lengths (expressed as the numbers of amino acids) are provided for circulating sequences for different time periods and subtypes or CRF. Kruskal-Wallis tests were used to compare the difference across time periods.

### Limited relationship between glycans and the development of neutralization breadth.

Neutralization assays were performed on samples collected up to 3 to 4 years after infection from both RV144 and RV217 participants to determine the percentage of viruses that could be neutralized in a panel of 34 viruses (37, 39, 40). We compared the number of glycans and loop lengths between sequences from individuals who developed either more than 70% or less than 35% breadth. Among 91 RV144 participants (74 with CRF01_AE infections sequenced), following 3 to 4 years of infection, 8 (7 with CRF01_AE sequence data) developed 70% breadth, and 23 showed less than 35% breadth (16 with CRF01_AE sequence data) (39). Among 68 RV217 participants (65 with sequence data), 13 developed 70% breadth, and 12 showed less than 35% breadth (40).

**FIG 6:**
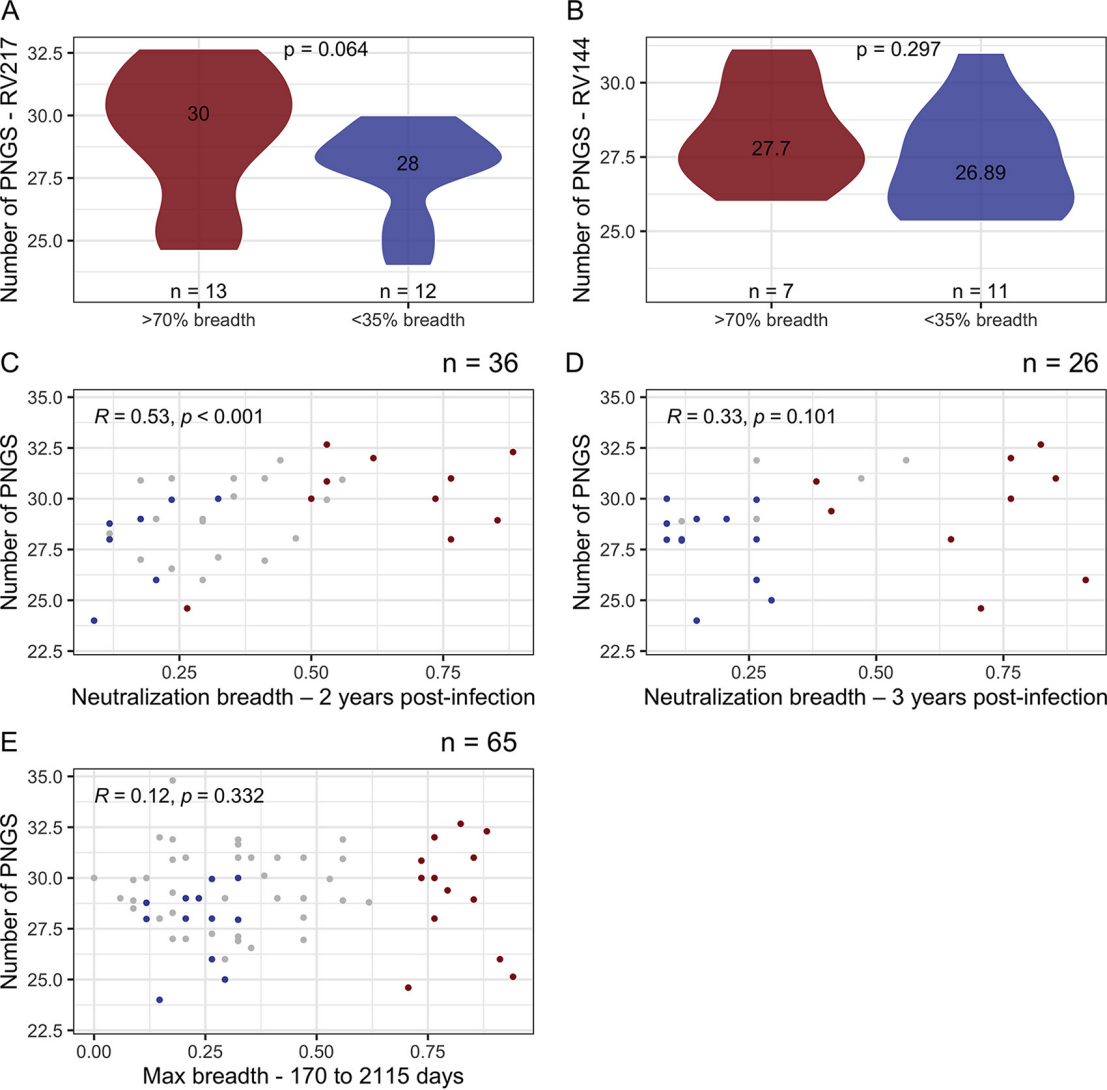
Relationship between number of PNGS and neutralization breadth. The number of PNGS identified in Env sequences during acute HIV-1 infection in RV217 (A) and at diagnosis in RV144 (B) participants was compared across participants who developed neutralization breadth (greater than 70% of the 34-virus panel, in red) or not (<35% of the 34-virus panel, in blue). The figures show the number of participants and median values for each group, along with *P* values for Mann-Whitney tests. The relationship between numbers of PNGS during acute infection and neutralization breadth measured 2 years (C), 3 years postinfection (D), or at peak breadth (E) was calculated using Spearman’s correlations, including all participants’ data in RV217. The correlation between increased breadth for Env with high numbers of PNGS observed 2 years after infection was not seen 3 years after infection or at peak breadth. The peak breadth measurements occurred between 170 and 2,115 days after diagnosis. The figures show Spearman’s ρ and *P* values, along with the number of individuals in each subgroup.

Among RV217 participants, there were more PNGS in sequences from individuals who developed bNAbs (*n* = 30) than in those who did not develop bNAbs over time (*n* = 28), and yet this difference did not reach statistical significance: *P* = 0.064 for sequences sampled in acute infection (Fig. 6A). We found evidence of a relationship between the number of PNGS and the development of neutralization breadth 2 years after infection. Specifically, breadth was positively associated with all the PNGS metrics in acute infection (Rho = 0.53, *P* = 0.001) (Fig. 6C). However, this relationship disappeared when breadth was measured 3 years after infection (Rho = 0.33, *P* = 0.101) (Fig. 6D) or at peak breadth (Rho = 0.12, *P* = 0.332) (Fig. 6E).

Considering the RV144 data set, contrary to the RV217 data set, we found no evidence that individuals who developed breadth had more PNGS: there was a median of 28 PNGS in sequences from individuals who developed bNAbs, and there was a median of 27 in those who did not develop bNAbs over time (*P* = 0.297) (Fig. 6B). There was no evidence of an association between the number of PNGS in early infection (within 6 months) and the development of neutralization breadth 3 years after infection in placebo participants (Rho = −0.058, *P* = 0.754). This may be due to the fact that only eight placebo recipients developed breadth in this cohort. In contrast, in vaccinees, an increase in the number of PNGS was associated with increased neutralization breadth (Rho = 0.57, *P* = 0.027); however, only two vaccinees showed bNAbs that could neutralize more than half of the virus panel. Similarly, there was no association between Env variable loop lengths and the development of neutralization breadth in both the RV217 and RV144 cohorts (*P* ≥ 0.254).

### Modeling the glycan shield.

As the number of PNGS and loop lengths are not the best representation of the glycans covering the envelope, we investigated computational models of the glycan shield that account for the resolved Env structure. We adapted the method developed by Wagh et al. (30) and added two features. First, we replaced the all-or-none shielding distance cutoff of 10 angstrom (Å) with a function centered ∼12 Å (0.5 Å). By doing this, the shielding probability was set to gradually decline between 6 Å (1 Å) and 18 Å (0 Å). Second, to calculate the hole area H, we used the number of surface atoms instead of the accessible surface area (ASA) for sites that lacked a well-defined structure such as the hypervariable sites in the loops (Wagh et al. defined the hole area [H_w_] as the sum of the ASA of the sites in the glycan hole). Our implementation showed results (Rho = −0.66, *P* = 0.019) similar to those of the original study by Wagh et al., which we could reproduce (Rho = –0.65, *P* = 0.023).

**FIG 7:**
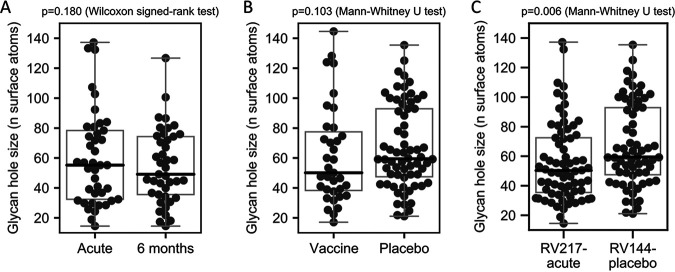
Variation in Env glycan hole size across early HIV-1 infection cohorts. The glycan hole size was compared across cohorts. (A) 41 RV217 participants with sequences sampled in the first month after diagnosis and 6 months later; (B) RV144 vaccine (*n* = 35) and placebo (*n* = 66) participants; (C) RV217 participants with sequences sampled in the first month after diagnosis (*n* = 69) and RV144 placebo participants (*n* = 66). The *P* values and corresponding statistical tests are listed above each panel.

We calculated the size of the glycan hole in our sequences. In the RV217 data set, using sequences sampled in acute infection (within 42 days of diagnosis) the glycan hole had a median size H = 55.2 surface atoms (range, 14.5 to 137.2). Results were similar using sequences sampled 6 months after infection: the glycan hole had a median size H = 49.1 surface atoms (range, 14.5 to 126.7) (Fig. 7A). Hence, there was no significant difference between glycan holes measures corresponding to sequences from the first month of infection or to sequences sampled 6 months later (*P* = 0.180, Wilcoxon signed-rank test) (Fig. 7A). Similar results were obtained when using the method developed by Wagh et al. (*P* = 0.815). In the RV144 data set, the median glycan hole size was 59.0 surface atoms (range, 17.1 to 144.6). The glycan hole size in sequences from RV144 placebo recipients tended to be slightly higher than the glycan hole size in vaccine recipients (*P* = 0.103) (Fig. 7B) and significantly higher than the glycan hole size in RV217 participants (*P* = 0.006) (Fig. 7C).

**FIG 8:**
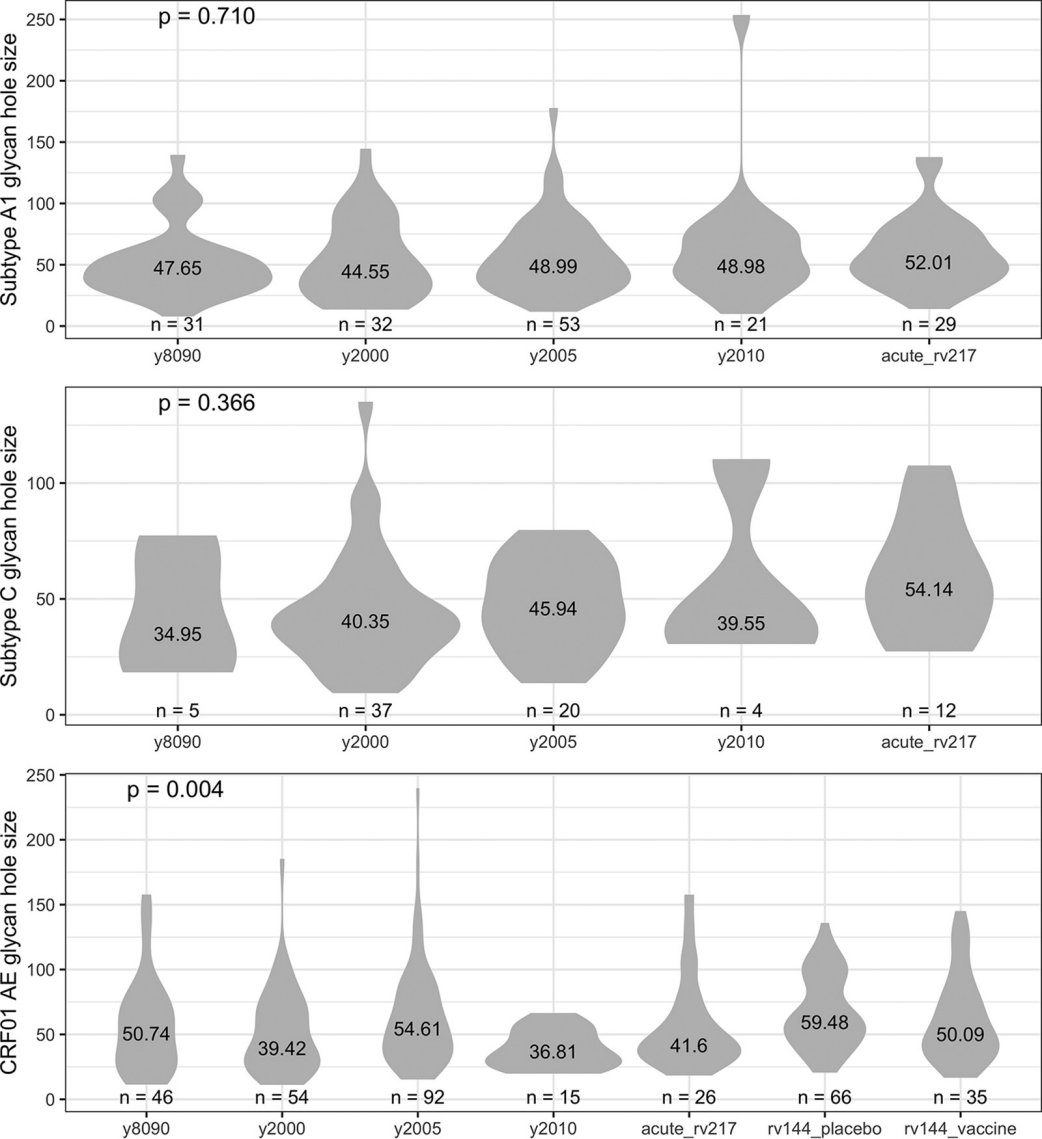
Variability in glycan hole sizes over time. The median glycan holes found in circulating Env sequences was compared over time and across subtypes. Data sets of circulating sequences were compared to the RV217 and RV144 cohorts; sequences from RV144 vaccine and placebo recipients were analyzed separately. The figure shows the numbers of participants and the median values for each group, along with *P* values for Kruskal-Wallis tests.

**FIG 9:**
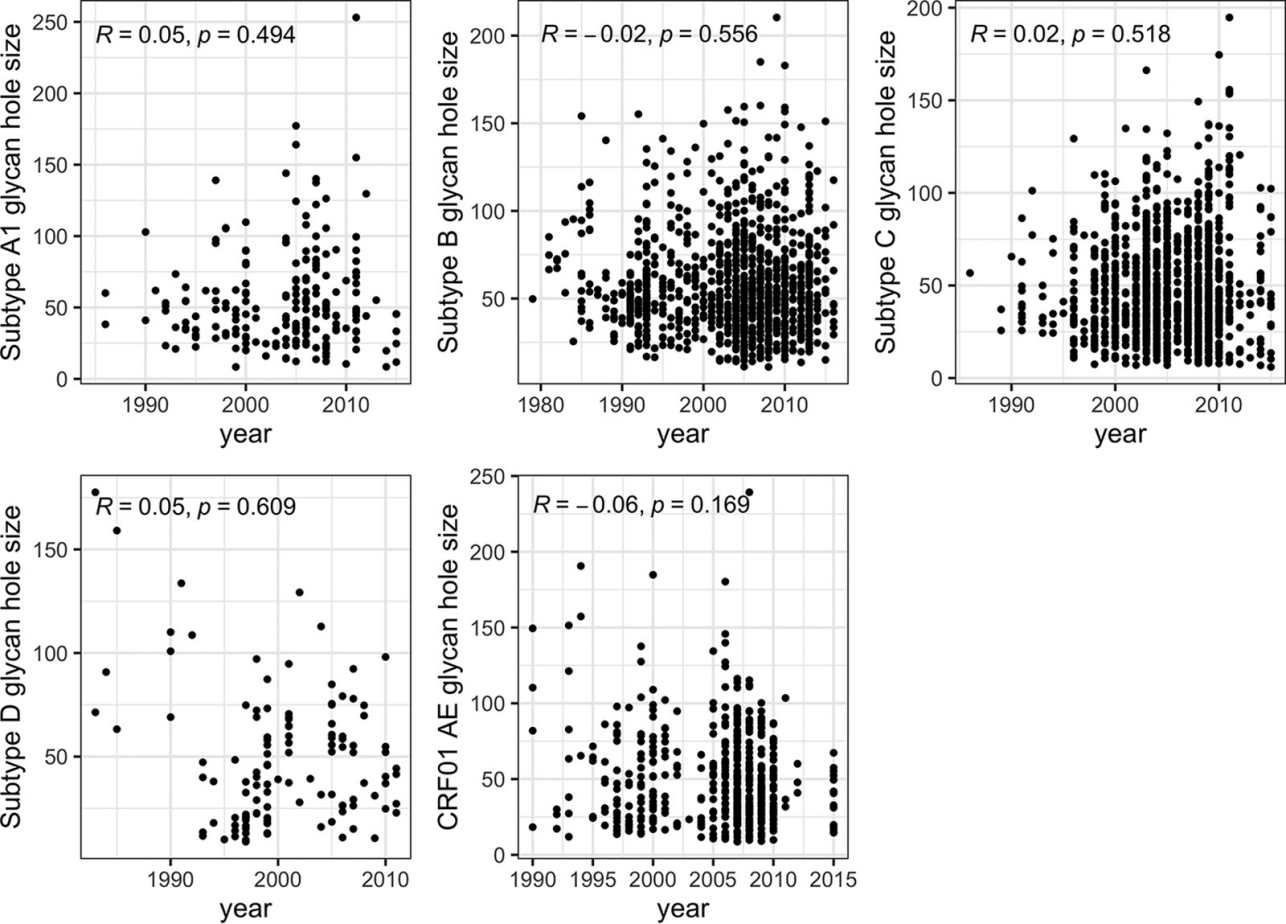
No variation in glycan hole sizes in Env sequences sampled since the beginning of the epidemic. The relationship between glycan holes and time was calculated using Spearman’s correlations. Similar patterns were seen across HIV-1 subtypes. The number of independent sequences sampled per subtype varied between 116 (subtype D) and 1184 (subtype C) sequences. The figure shows Spearman’s ρ and *P* values.

To evaluate whether sequences from RV217 and RV144 participants were representative of global HIV-1 sequences, we calculated glycan hole sizes in the large data sets of circulating sequences representing HIV-1 subtypes and CRF. In the past decade, median glycan hole sizes ranged between 42.0 surface atoms (subtype D, *n* = 116 sequences) and 51.7 surface atoms (subtype B, *n* = 1,035 sequences) across data sets (IQR = 32.8 to 45.5) (Fig. 8). Glycan holes from RV217 participants infected with subtypes A1 or C were comparable to those calculated in circulating Env sequences. Likewise, glycan holes in RV144 participants were similar to those found in circulating CRF01_AE sequences. Finally, we found no evidence that glycan holes calculated across 3115 Env sequences tended to decrease or increase since the beginning of the epidemic (Fig. 9).

### No relationship between the completeness of the glycan shield and the development of neutralization breadth.

**FIG 10:**
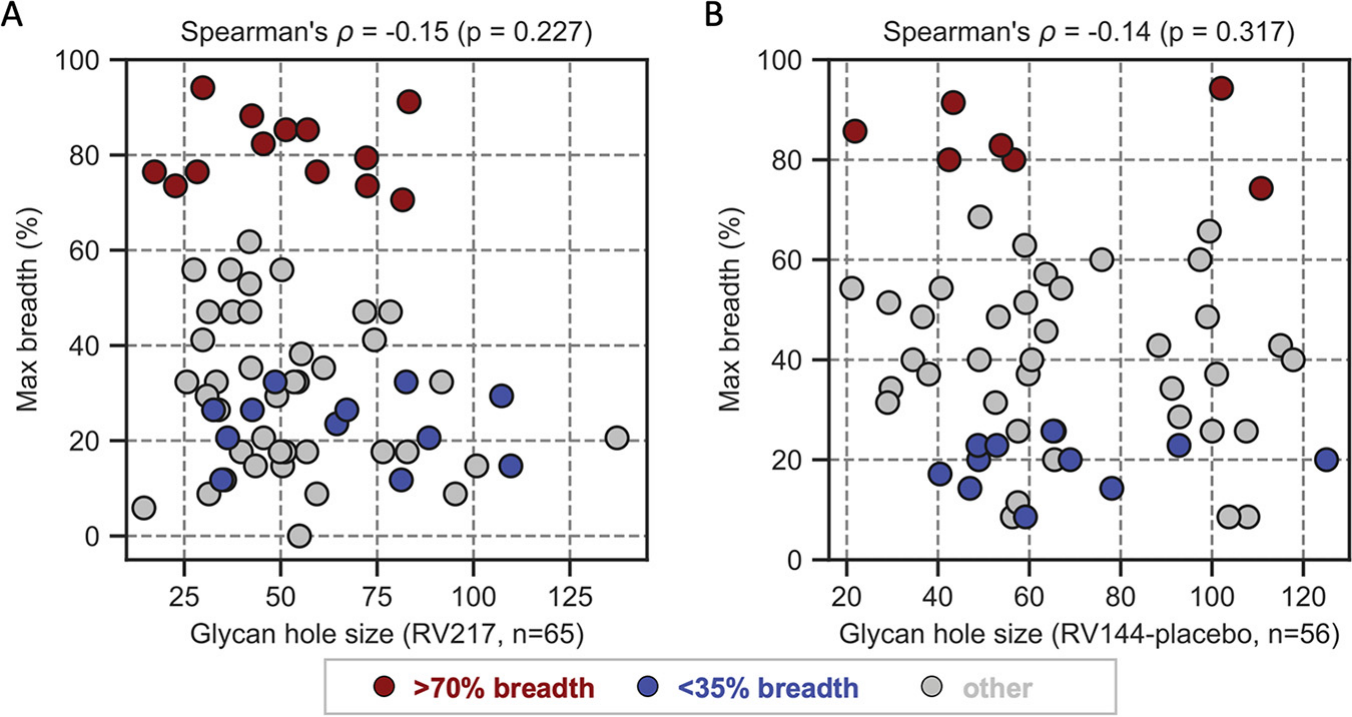
Lack of relationship between the glycan hole size and maximum neutralization breadth. HIV-1 Env sequences were obtained in acute infection from RV217 (A) and RV144 (B) participants; only RV144 placebo recipients were included. Neutralization breadth was assessed against a 34-virus panel, and participants were classified as having developed >70% (red), <35% (blue), or intermediate (gray) breadth.

We analyzed the relationship between glycan hole size and neutralization breadth. In the RV217 cohort, the founder hole size or area was smaller in the group of individuals who developed bNAbs (H = 51.4 surface atoms) than in those who did not develop breadth (65.8 surface atoms), but this difference was not significant (*P* = 0.091). We found no relationship between the glycan hole size and the neutralization breadth measured 3 years after infection (Rho = −0.13, *P* = 0.435) nor at 2 years (Rho = −0.12, *P* = 0.563). The results were similar using peak breadth measurements (Rho = −0.15, *P* = 0.227) (Fig. 10A). Since our neutralization assays were performed earlier in infection (at a median of 926 days postdiagnosis) than in the individuals reported in the study by Wagh et al. (median = 1,398 days), we censored data in the Wagh cohort to obtain a similar median as in RV217 (962 days). The original relationship reported by Wagh was maintained (Rho = –0.71, *P* = 0.010), indicating that the earlier data in our cohort was not the cause of the lack of relationship between glycan hole size and neutralization in RV217. Moreover, in the RV144 placebo group we also found no evidence of a relationship between small glycan holes and the development of neutralization breadth (Rho = −0.14, *P* = 0.317) (Fig. 10B). The results were similar when we included both vaccine and placebo groups (Rho = −0.10, *P* = 0.334).

## DISCUSSION

An ideal HIV-1 vaccine candidate would replicate early stages of HIV-1 infection within individuals who develop bNAbs to promote the development of potent neutralization breadth. We therefore investigated whether specific characteristics of the HIV-1 Env glycan shield were associated with the development of neutralization breadth. Specifically, we evaluated glycan shield features present in acute and early HIV-1 infections in multiple HIV-1 subtypes and compared these features to neutralization breadth measurements obtained 2 to 3 years after infection in 121 participants enrolled in two cohorts. Overall, our comprehensive analysis revealed limited evidence that supported HIV-1 acute or early infection glycan determinants that could robustly predict the subsequent development of neutralization breadth in these cohorts. This is consistent with the finding that the predicted neutralization specificities target principally the MPER and CD4bs rather than glycans in these cohorts (40). Collectively, our results emphasize that the development of neutralization breadth is a complex process involving the interaction of distinct viral and host immune characteristics overtime.

First, we studied the effect of standard measures of the HIV-1 Env glycan shield, such as the variable loop lengths or number of PNGS, on the development of neutralization breadth. Past studies had suggested that shorter variable loop lengths or lower number of PNGS could be advantageous toward the development of neutralization breadth (19, 23, 26, 29). However, we did not see evidence of such patterns in RV217 or RV144. Rather, although our results did not reach significance, it appeared that the direction of this trend was opposite: Env with longer loops and more glycans or denser glycan shields tended to be associated with the development of broader neutralizing responses. This positive relationship between glycans and neutralization breadth was previously reported in a large study (*n* = 389 Env) by Hraber et al. (43). Previous studies had shown that infections with subtype C were associated with greater development of neutralization breadth (17, 44). Participants in our study were infected with different subtypes, and yet we found no evidence of subtype-specific differences, possibly due to the small size of our cohorts; in addition, since none of the subtype C-infected individuals in our cohorts developed neutralization breadth, we could not assess subtype C-specific effects. A strength of our study was that we conducted analyses in two independent cohorts in two disparate geographic regions in which both the sequence and neutralization data were obtained in the same lab using the same methodology. We analyzed 65 participants from a prospective acute infection cohort, RV217, and 56 breakthrough infections that occurred during the study period for a vaccine efficacy trial, RV144. It is possible that our study size reflects a regression toward the mean phenomenon, whereby results previously identified in smaller cohorts could not be reproduced, and yet our study size was too small to evidence factors of small effect sizes.

Second, we used a more sophisticated method to describe the glycan shield than the glycan or Env lengths measures. This method was originally developed by Wagh et al. (30), and we included two additional features to better account for the sites that lack a well-defined conformation in known Env structures and to implement a gradual distance threshold to the area shielded by a given glycan. This method allows estimation of glycan hole size and, conversely, yields an estimate of the completeness of the glycan shield. Recent studies showed that glycan holes are associated with the development of strain-specific neutralizing responses, via the targeting of the Env epitope unmasked due to the proximity of a glycan hole (31, 32, 45, 46). Additional studies showed that this glycan hole effect could be engineered and reproduced for different neo-epitopes, yielding distinct neutralization specificities (47–51). However, the unmasking of different glycan holes did not yield an increase in cross-reactive responses (33, 34). Wagh et al. showed that there was a significant positive relationship between the completeness of the glycan shield (or smaller glycan holes) and the development of neutralization breadth in a cohort of 12 individuals (30). In contrast, there was no evidence that more complete glycan shields were associated with subsequent breadth in our RV217 and RV144 cohorts, despite our larger sample size (121 individuals). We verified that the lack of significance was not due to differences in the time of the neutralization breadth assessments. Nonetheless, we note that when we compared the extremes of the distribution in our cohorts, the glycan shield tended to be more dense in individuals who later developed neutralization breadth than in those with restricted development of breadth 3 years after infections, although this difference did not reach significance (*P* = 0.091). Likewise, Env sequences from these participants tended to include more predicted glycans than Env from the individuals who did not develop breadth (*P* = 0.064). While a complete glycan shield in early infection was not sufficient to predetermine the subsequent development of neutralization breadth, further longitudinal characterization of glycan shield dynamics may help determine whether specific glycan shield rearrangements could promote neutralization breadth.

Third, our results were corroborated by an analysis of publicly available circulating HIV-1 sequences. The analysis of over 3,000 independent HIV-1 Env sequences sampled since the beginning of the epidemic revealed no significant evolution in the number of PNGS or the size of the loop lengths over time. Likewise, glycan holes showed large variability across circulating sequences (IQR = 32.8 to 45.5), yet there was no evidence of a trend toward an increase or a decrease over time (correlations with the year of sampling ranged from −0.06 to 0.05). Together with our cohort-specific findings, these results indicate that there is no evidence of a global selective pressure that would act unidirectionally on HIV-1 sequences toward optimal glycan features. The dense arrangement of glycans allows to shield HIV-1 from the host immune recognition and can afford escape from elimination by the host immune system (9, 52, 53). However, the lack of directional change we observed over time reflects that there is no pressure to evolve toward a lower versus higher number of glycans or a more complete glycan shield. These findings indicate that evolutionary pressures on the glycan shield reflect a complex set of interactions, where opposite forces can act. In an infected individual, the appearance of a glycan hole followed by its filling may lead to a repeated process that could shift across epitopes, as observed for the glycans at sites 332 and 334 (54). Additional studies are needed across larger cohorts to evaluate glycan dynamics longitudinally at the individual level and to explain how individual patterns translate at the population level.

In conclusion, our comprehensive analysis indicated that the development of neutralization breadth in HIV-1-infected participants in the RV217 and RV144 cohorts could not be ascribed to any single HIV-1 Env glycan shield feature such as the loop lengths, the number of PNGS, or the completeness of the glycan shield. Our findings illustrate that the development of neutralization breadth is a continuous, prolonged process where a cycle of viral mutations and host immune responses create multiple paths toward improved neutralization, emphasizing that glycan shield characteristics do not act in isolation from other parameters.

## MATERIALS AND METHODS

### Sequence data set.

RV144 and RV217 HIV-1 genome sequences were derived via single genome amplification from plasma samples and previously reported (37, 38). HIV-1 circulating sequences (one sequence per individual) were retrieved from Los Alamos HIV Sequence Database LANL-db. Sequences with no time stamp or subject identifier were excluded. A set of HIV-1 sequences representative of the worldwide distribution of HIV-1 group M sequences (55) was used as a reference for glycan hole size calculation. Sequences corresponding to the env gene were extracted and aligned using Gene Cutter at LANL-db (https://www.hiv.lanl.gov/content/sequence/GENE_CUTTER/cutter.html) and edited manually with AliView (56). Sequences with evidence of hypermutation were removed using Hypermut (https://www.hiv.lanl.gov/content/sequence/HYPERMUT/hypermut.html).

Nucleotide sequences were translated to amino acid (aa) sequences. For sites with ambiguities that coded for different aa, if one of the aa corresponded to the consensus of all the sequences the given participant, this residue was chosen (otherwise “X” was used). Sequences with frame shifts or stop codons were removed except if the frameshift or stop codon was located at the N- or C-terminal codon of Env. Twenty-one sequences from 15 participants were excluded from the analysis because they presented one or more deletions at least 3 aa in length in conserved regions and were not observed in any other sequences in the alignment.

### Neutralization data.

Results from the neutralization assay were previously reported for R217 and RV144 participants (39, 40). Briefly, plasma samples collected prior to ART initiation were tested against 34 viruses representing subtypes A, B, C, D, CRF_01 AE, and CRF_02 AG. The positivity criteria were as follows: 50% inhibition of infection of a given HIV-1 virus at ≥1:40 plasma dilution, twice the limit of detection for the assay, and less than 50% inhibition of infection of murine leukemia virus. Breadth was calculated as the percentage of the 34-virus panel neutralized by ≥1:40 plasma dilution. Potency was calculated by the geometric mean titer of the 50% inhibitory dilution of all viruses in the panel. Neutralization data from 95 RV144 participants were available, and samples from four RV144 vaccine recipients who did not receive all immunizations were excluded from analysis. Neutralization data from 68 RV217 participants were available.

### PNGS predictions.

The 4-aa N !P S/T !P pattern was used to identify potential N-linked glycosylation sites for each sequence. Five or more sequences per participant per time point were used to calculate the mean number of PNGS and the mean loop length.

### Estimation of the glycan hole size.

Env sites that were considered accessible to antibodies were defined based on the resolved closed prefusion Env trimer structure (PDB code 5FYJ) (57) as those with a relative accessible surface area (ASA) (58) greater than 0.08. The relative ASA was calculated as the ratio of the side chain ASA (scASA; where C_α_ are included) over the maximum ASA (maxASA) of a residue (59). The residue depth, which corresponds to the distance from the residue to the solvent, was calculated using the DEPTH webserver (http://cospi.iiserpune.ac.in/depth/) (60). Two depth definitions were used to exclude surface sites in cavities of different sizes. For large cavities, such as the center of the closed prefusion trimer, the parameter “neighborhood waters” was set to its maximum value (= 5) to calculate the depth (depth_5HOH_). Surface sites with depth_5HOH_  > 8 Å were excluded. For small cavities, the parameter “neighborhood waters” was set to its default value (= 2) to calculate the depth (depth_2HOH_). Surface sites with depth_2HOH_  > 4.5 Å were excluded.

Additional sites were manually adjusted after visual inspection of the Env structure: sites 162, 166, 175, 299, 322A, 364, 411, 419, 456, and 458 were originally misclassified by the automatic procedure but were considered as accessible, while sites 482 and 502 to 506 (bottom of the prefusion Env trimer) were excluded as they were not accessible on viral particles.

Each sequence was mapped onto the 5FYJ structure to extract accessible surface sites. Sites that corresponded to an insertion with respect to 5FYJ or were located in a hypervariable segment were considered as accessible surface sites. Sites located beyond the C-terminal and N-terminal of 5FYJ were not included as accessible surface sites. The probability of a surface site i to be shielded was estimated according to its distance to all predicted PNGS by:

(1) $$P_{i}=1-\Pi_{j}(1-P_{\mathrm{ij}})$$

(2) $$P_{\mathrm{ij}}=1/(1+e^{d_{\mathrm{ij}}-12.0})$$

where *P_i_* is the probability that site *i* is shielded, *P_ij_* is the probability that site *i* is shielded by PNGS *j*, and *d_ij_* is the distance between the C_β_ atoms of sites *i* and *j*. For insertions or sites in the hypervariable segments (for which the distance cannot be directly predicted from 5FYJ), a pseudo distance is estimated. The glycan hole size is calculated as:

(3) $$\text{Hole size}=\Sigma_{i}(P_{i\_\mathrm{ref}}-P_{i})s_{i}$$

where *P_i_ref_* is the baseline probability of site *i* (the mean probability that a site is shielded in a set of sequences representative of HIV-1 group M), *s_i_* is the number of nonhydrogen side chain atoms of the amino acid on site *i* (C_α_ was considered as a side chain atom to give one surface atom for surface Gly), and the summation is over all surface sites with *P_i_ref_*  > 0.5 and *P_i_ref_* − *P_i_* > 0.1. The glycan hole size for sequences from a given participant at a time point was defined as the mean glycan hole size of all the sequences available from the participant at that time point.

### Statistical analysis.

Visualization and statistical testing were performed in Python (Scipy was used for the calculation of Spearman’s correlation, as well as pairwise comparison of glycan hole area and glycan hole size [61]) and R (with R packages ggplot2 [62]), ggpubr (https://rpkgs.datanovia.com/ggpubr/), and GGally (https://github.com/ggobi/ggally).
